# Advancing Diabetic Foot Ulcer Care: AI and Generative AI Approaches for Classification, Prediction, Segmentation, and Detection

**DOI:** 10.3390/healthcare13060648

**Published:** 2025-03-16

**Authors:** Suhaylah Alkhalefah, Isra AlTuraiki, Najwa Altwaijry

**Affiliations:** Computer Science Department, College of Computer and Information Sciences, King Saud University, Riyadh 11543, Saudi Arabia; ialturaiki@ksu.edu.sa (I.A.); ntwaijry@ksu.edu.sa (N.A.)

**Keywords:** diabetic foot ulcers, artificial intelligence, machine learning, generative AI, mobile applications

## Abstract

**Background**: Diabetic foot ulcers (DFUs) represent a significant challenge in managing diabetes, leading to higher patient complications and increased healthcare costs. Traditional approaches, such as manual wound assessment and diagnostic tool usage, often require significant resources, including skilled clinicians, specialized equipment, and extensive time. Artificial intelligence (AI) and generative AI offer promising solutions for improving DFU management. This study systematically reviews the role of AI in DFU classification, prediction, segmentation, and detection. Furthermore, it highlights the role of generative AI in overcoming data scarcity and potential of AI-based smartphone applications for remote monitoring and diagnosis. **Methods**: A systematic literature review was conducted following the PRISMA guidelines. Relevant studies published between 2020 and 2025 were identified from databases including PubMed, IEEE Xplore, Scopus, and Web of Science. The review focused on AI and generative AI applications in DFU and excluded non-DFU-related medical imaging articles. **Results**: This study indicates that AI-powered models have significantly improved DFU classification accuracy, early detection, and predictive modeling. Generative AI techniques, such as GANs and diffusion models, have demonstrated potential in addressing dataset limitations by generating synthetic DFU images. Additionally, AI-powered smartphone applications provide cost-effective solutions for DFU monitoring, potentially improving diagnosis. **Conclusions**: AI and generative AI are transforming DFU management by enhancing diagnostic accuracy and predictive capabilities. Future research should prioritize explainable AI frameworks and diverse datasets for AI-driven healthcare solutions to facilitate broader clinical adoption.

## 1. Introduction

Diabetes mellitus (DM) is a chronic condition that requires regular attention and concern for many aspects besides blood sugar levels [1]. Currently, several people are suffering from diabetes all over the world, and estimates suggest that this number will exceed 700 million globally before the year 2050 [2]. Diabetic foot ulcer (DFU) is one of the most common complications related to diabetes mellitus and one of the leading factors for mortality rates and healthcare costs [3]. According to worldwide estimates, up to 25% of people with diabetes are likely to develop a foot ulcer sometime in their lives [4]. Besides causing pain, such ulcers might also progress into severe infections and gangrene, culminating in lower limb amputation in many instances [5].

Traditional DFU care includes regular clinical evaluations, wound cleaning, and surgical interventions when necessary. However, these methods are often resource-intensive and require specialized expertise [6]. Additionally, the rapid progression of DFUs necessitates early diagnosis and intervention to prevent severe complications [7]. Recent advances in medical technologies, such as artificial intelligence (AI) [8] and generative AI [9], help address these challenges. AI refers to using computer systems to perform tasks that typically depend on human cognitive abilities, such as analyzing medical images. John McCarthy coined the term and defined it as “the science and engineering of making intelligent machines, especially intelligent computer programs” [10]. Generative AI, a subset of AI, focuses on creating new data based on existing patterns [11]. These advancements enhance diagnostic accuracy, optimize care processes, and improve patient outcomes.

Several review studies have explored the application of AI in tasks related to DFU management [12], including classification, segmentation, detection, and predictive modeling. For instance, prior reviews highlight how convolutional neural networks (CNNs) are increasingly adopted for DFU classification [13,14,15]. Similarly, prior reviews have highlighted the use of state-of-the-art method models, including machine learning algorithms such as support vector machines (SVMs) [16,17], and artificial neural networks (ANNs) [18,19] to forecast DFU healing outcomes and amputation risks. Segmentation techniques leveraging CNN architectures, such as UNet and LinkNet [20], and Double Encoder-ResUNet (DE-ResUNet) [21] have demonstrated significant progress in delineating DFU boundaries. Researchers have employed advanced DL architectures like EfficientNet [22] and DenseNet [23] to enhance the detection of DFUs in clinical and telemedicine settings. They have utilized models such as conditional generative adversarial networks (cGANs) [24] and diffusion models [25] to generate synthetic datasets for DFU research in the context of generative AI.

While these reviews provide valuable insights into advancements in technical innovations, they predominantly focus on algorithmic performance metrics, such as accuracy, precision, recall, and F1 scores, often overlooking the practical integration of AI into real-world applications, including mobile health technologies. However, several individual studies have highlighted the transformative potential of AI-powered smartphone applications; such solutions enable advancements in areas such as predictive healing, monitoring, wound localization, and detection, displaying the practical utility of these technologies in diverse clinical settings.

To address this gap, this paper aims to review the integration of AI in DFU management, focusing on real-world applicability. The specific contributions of this paper are as follows: (i) It provides review of the integration of AI in the classification, prediction, and segmentation of DFUs. (ii) It explores the role of generative AI in enhancing data augmentation and addressing limitations in dataset availability. (iii) It conducts an examination of innovations in mobile application technologies for the monitoring and management of DFUs.

## 2. Methods

This review followed the Preferred Reporting Items for Systematic reviews and Meta-Analyses (PRISMA) [26]. The review protocol was registered at the International Platform of Registered Systematic Review and Meta-analysis Protocols (INPLASY) [27] under the registration number INPLASY202520066 (doi: 10.37766/inplasy2025.2.0066).

The method used to conduct this research is as follows: First, the research questions are described, followed by the inclusion and exclusion criteria. The search strategy is then stated; finally, the study selection process is presented.

### 2.1. Research Questions

This systematic review aimed to answer the following research questions:

RQ1: What are the roles of AI in managing DFU?

RQ2: What role do generative AI techniques play in overcoming challenges?

RQ3: What are the major challenges and future directions in using AI for DFU management?

### 2.2. Inclusion and Exclusion Criteria

The inclusion criteria are as follows:

- Articles focused on the application of AI or generative AI in DFU;
- Article published during the period from 2020 to 2025;
- Studies in the English language.

The exclusion criteria are as follows:

- Articles focused on non-DFU-related medical imaging or conditions;
- Articles unrelated to AI applications in DFUs.

### 2.3. Search Strategy

Researchers conducted the search strategy across multiple databases, including PubMed, IEEE Xplore, Google Scholar, Scopus, and Web of Science, to comprehensively retrieve relevant studies. They used a combination of keywords such as “diabetic foot ulcers”, “artificial intelligence”, “machine learning”, “generative AI”, “mobile applications”, “classification”, “prediction”, “segmentation”, and “detection”. To refine the search results and ensure thorough coverage of the literature, they applied Boolean operators (AND/OR).

### 2.4. Study Selection

**Figure 1:**
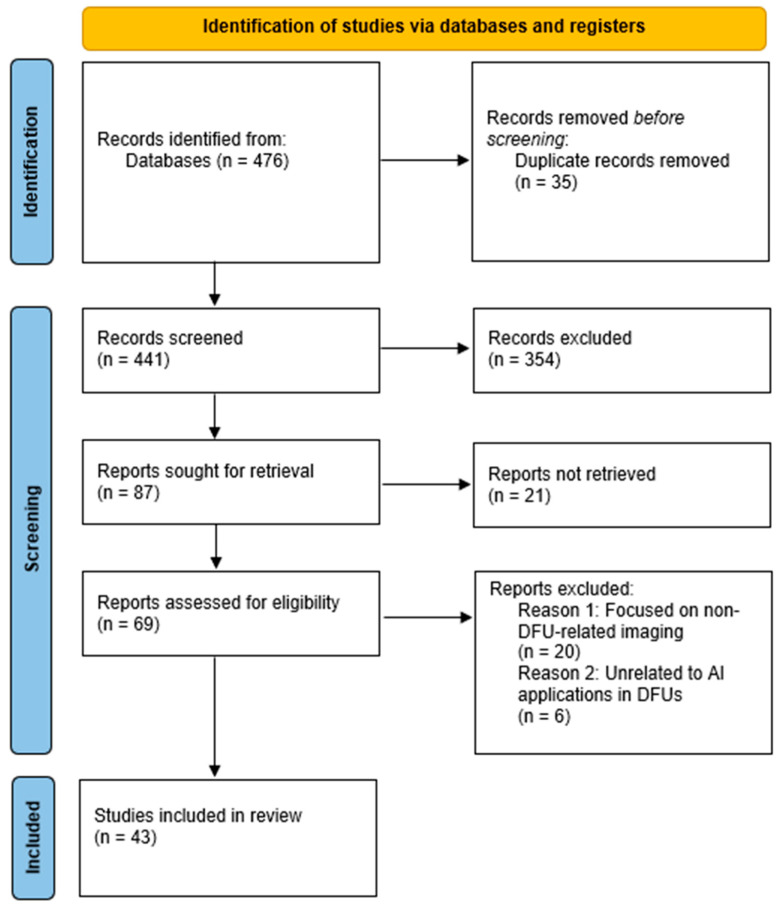
PRISMA flow diagram.

The initial search phase retrieved a total of 476 studies. Researchers uploaded the references to EndNote X8 [28], where they identified and removed duplicate records, reducing the number of studies to 441. They then screened these studies based on their titles and abstracts, excluding 354 studies irrelevant to DFU research or classified as review articles rather than original research. This process left 87 studies for full-text retrieval; however, they could not retrieve 21 reports. Following this screening process, researchers assessed 69 full-text studies for eligibility. They applied the predefined inclusion and exclusion criteria, leading to the exclusion of 26 studies. Ultimately, they selected 43 studies for full-text review. Researchers documented the entire process using a PRISMA flow diagram, which outlines the number of studies retrieved, screened, excluded, and included in the systematic review, as shown in Figure 1.

## 3. Results

**Figure 2:**
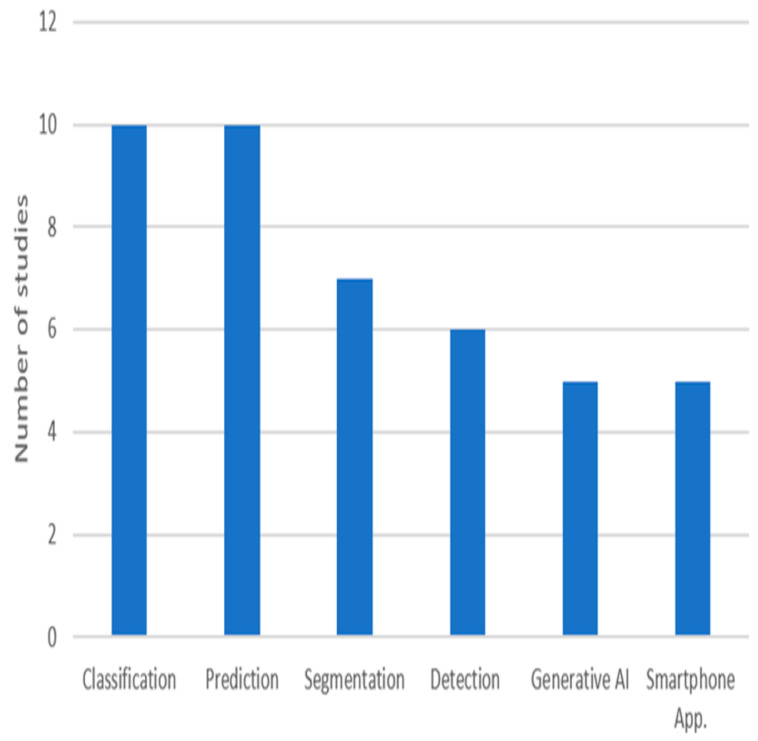
The purposes of the reviewed studies.

The integration of advanced technologies has become critical in addressing the complexities of DFUs, which remain a significant challenge to healthcare systems worldwide. Traditional DFU management methods, such as visual inspection and manual measurement, often produce variable outcomes due to differences in clinical expertise. With the growing incidence of diabetes, there is a need for innovative solutions that enhance diagnostic precision and reduce the risk of complications such as infections and amputations. Emerging digital solutions, particularly those powered by AI, have the potential to revolutionize DFU management by providing effective tools. AI-based techniques enable clinicians to achieve higher diagnostic accuracy and offer personalized care plans, even in resource-limited settings characterized by data scarcity and shortages in human resources. This section aims to explore how AI has transformed DFU-related tasks, including classification, prediction, segmentation, and detection, and to discuss its practical application in clinical practice for improving patient care. Figure 2 shows the number of reviewed studies for each task. Among these, classification and prediction have received the most attention in research, highlighting their fundamental role in DFU care.

### 3.1. Classification

Alzubaidi et al. [13] introduced DFU_QUTNet, a deep convolutional neural network (CNN), for classifying DFUs into healthy or abnormal skin categories. The researchers collected the dataset from a hospital in Iraq, capturing both healthy (normal) and DFU-affected skin (abnormal). The researchers augmented the images using rotation, flipping, and contrast adjustments to address the dataset’s small size. Unlike traditional convolutional neural networks, where increasing the depth by stacking more layers can degrade performance due to issues like those related to gradient, DFU_QUTNet is designed with a wider network while maintaining depth, ensuring better gradient propagation. The DFU_QUTNet architecture incorporates 17 convolutional layers, batch normalization, Rectified Linear Unit (ReLU) activations, and global average pooling, resulting in robust feature extraction, such as edges. DFU_QUTNet outperformed GoogleNet, VGG16, AlexNet, and DFUNet [29]. Additionally, SVM outperformed k-nearest neighbor (KNN) when combined with DFU_QUTNet.

Amin et al. [30] introduced a deep learning framework for the classification and localization of DFUs, focusing on two complications such as infection (bacteria in the wound) and ischemia (inadequate blood supply to the affected area). The framework employs a 16-layer CNN, which includes three convolutional layers, three batch layers, three ReLU layers, one average pooling layer, one skip convolutional layer, one addition layer, one fully connected layer, one softmax layer, and one classification output layer, enabling effective feature extraction and classification. For localization, the CNN is paired with the YOLOv2-DFU model, built on YOLOv2 with ShuffleNet as a backbone. The researchers evaluated the model’s performance using the DFU-Part (B) dataset [31]. The data were augmented to enhance model robustness. Classification experiments employed multiple machine learning classifiers, with the Decision Tree (DT) and Naive Bayes (NB) achieving the highest accuracy for infection and ischemia. After classification, gradient-weighted class activation mapping was utilized to validate the model’s decisions.

Ahsan et al. [15] addressed the classification of DFU ischemia and infection using the DFU2020 dataset [32]. The dataset was expanded through augmentation techniques like rotation, flipping, scaling, translation, noise addition, and shearing. Transfer learning with fine-tuning was employed to overcome the challenges of limited medical image datasets, enabling effective DFU classification. Several CNN architectures were evaluated, including AlexNet, VGG16/19, GoogLeNet, ResNet50/101, MobileNet, SqueezeNet, and DenseNet. ResNet50 achieved the best performance due to its skip connections, which allowed for deeper network training and mitigated vanishing gradient issues. This approach highlights the effectiveness of combining transfer learning with a robust CNN architecture for addressing challenges in small, specialized medical datasets.

Toofanee et al. [33] introduced DFU-SIAM, a deep learning model, for classifying DFUs using a Siamese Neural Network (SNN) architecture that integrates EfficientNetV2S (CNN) and BEiT (Vision Image Transformer) for feature extraction. Researchers trained the DFU-SIAM model on the DFUC2021 dataset [34], containing four classes for training: none, infection, ischemia, and both. This severe class imbalance is addressed through data augmentation techniques, including color jitter, random horizontal and vertical flips, and equalization, creating a more balanced training dataset. The Siamese architecture comprises two identical networks, extracting features from pairs of input images for similarity learning. EfficientNetV2S extracts detailed convolutional features, while BEiT captures global image contexts through masked image modeling. The model flattens, concatenates, and passes the outputs from both subnetworks to a classification layer. A KNN classifier is incorporated for final predictions, with the optimal number of neighbors determined dynamically based on the maximized Macro F1 score during training. DFU-SIAM leverages deep learning and network design to the DFU classification across multiple categories.

Liu et al. [14] presented a system for classifying infection and ischemia in DFUs using the EfficientNet deep learning architecture. It utilizes the publicly available DFUC2021 dataset [34], and unlike previous studies that attempted multi-class classification, this work simplifies the task into two binary classifications: infection (yes/no) and ischemia (yes/no). EfficientNet models from B0 to B7 variants were fine-tuned using transfer learning, where the early layers pre-trained on ImageNet were retained to extract generic features, and later layers were retrained to focus on specific wound features. The researchers used baseline models, including ResNet50, DenseNet121, Inception V3, and VGG16, for comparison. EfficientNet demonstrated superior performance and was significantly faster, completing tasks in 10–50% of the time required by baseline models, making them practical for real-time use.

Khalil et al. [35] presented a deep learning-based model for classifying diabetic foot sores (DFSs), specifically targeting abrasion foot sores (AFSs) and ischemic diabetic foot sores. They utilized two publicly available datasets, one from the Kaggle repository [36] and another from Alzubaidi et al. [13]. To overcome data scarcity, data augmentation techniques such as flipping, zooming, and noise reduction were used to expand the dataset to 8478 images. The authors proposed an architecture combining a pre-trained VGG-19 model for feature extraction and a custom six-layer CNN for classification. Image segmentation was performed using UNet++, a densely connected U-shaped architecture that enhances feature map resolution and minimizes segmentation errors, which is critical for accurately delineating lesion boundaries. Comparisons were made with models such as Inception-v3 and MobileNet, with the proposed model outperforming these baselines in all metrics. Furthermore, statistical analysis using ANOVA and Friedman tests validated the model’s robustness.

Alqahtani et al. [37] proposed a deep learning-based system to classify DFUs as normal or abnormal using the Adaptive Weighted Sub-Gradient Convolutional Neural Network (AWSg-CNN). The dataset was sourced from Kaggle [36]. Researchers preprocessed the data by removing missing or inconsistent entries and splitting the dataset into 80% training and 20% testing subsets. The AWSg-CNN integrates two key components: the random initialization of weights (RIW) and the Adaptive Sub-gradient Optimizer (ASGO). RIW helps prevent overfitting and ensures effective learning by adapting to high-dimensional data, while the ASGO stabilizes gradients and optimizes convergence rates through a log softmax function. The model achieved high classification accuracy, precision, recall, and F1 scores.

Preiya et al. [38] proposed a deep learning framework for analyzing foot ulcer images in diabetic patients, with a focus on classification and feature extraction. It uses the DFUC2021 dataset [34]. They integrated a Deep Recurrent Neural Network (DRNN) for feature extraction from numerical and text data with a Pre-trained Fast Convolutional Neural Network (PFCNN) integrated with U++Net for image classification. The model processes preprocessed and segmented data to distinguish between normal and abnormal diabetes ranges.

Fadhel et al. [39] proposed real-time classification models for distinguishing normal and abnormal DFUs using deep learning techniques combined with hardware acceleration platforms. The study utilized a dataset sourced from the Kaggle repository [36], where data augmentation was applied to address class imbalance. Two CNN models, DFU_FNet and DFU_TFNet, were introduced, each designed to optimize feature extraction and mitigate the limitations of small datasets. DFU_FNet employs a simpler architecture to extract features for training classifiers like SVM and KNN, while DFU_TFNet utilizes a deeper architecture enhanced with transfer learning, improving performance on medical imaging tasks. Both models were implemented on field-programmable gate arrays (FPGAs) and graphics processing units (GPUs). DFU_TFNet achieved remarkable results, outperforming models such as AlexNet, VGG16, and GoogleNet. The FPGA platform, while slightly slower in processing than GPUs, demonstrated significantly lower power consumption, making it an ideal candidate for portable, real-time diagnostic applications.

Gudivaka et al. [40] presented a machine learning (ML) approach for DFU classification, utilizing reinforcement learning (RL). It integrates compositional pattern-producing networks (CPPNs) for recognizing structured and unstructured images, SVM for classification, hierarchical clustering for grouping data, and ELM with a single hidden layer for fast classification. Additionally, they evaluated deep learning models, including AlexNet, VGG16, GoogLeNet, and ResNet50, on the DFU2020 dataset [32], with ResNet50 achieving the highest classification accuracy of 99.49% for ischemia detection. Data augmentation techniques such as affine transforms were applied. The results demonstrate significant improvements in classification efficiency, making this approach a promising advancement in DFU diagnosis. According to the clustering scenario analysis of DFU, the classification efficiency varies based on ulcer severity levels, with the model effectively distinguishing between four clusters: Cluster One: Mild to Moderate Localized Cellulitis; Cluster Two: Moderate to Severe Cellulitis; Cluster Three: Moderate to Severe Cellulitis with Ischemia; and Cluster Four: Life- or Limb-Threatening Infections.

Table 1 summarizes recent studies, highlighting AI models, data types, and performance metrics used for DFU classification.

### 3.2. Prediction

Lin et al. [41] aimed to establish prediction models for evaluating the amputation and survival probabilities in patients with diabetic foot (DF). The dataset comprised 200 inpatients. Researchers included patients who exhibited clinical symptoms of DF, such as impaired circulation and peripheral neuropathy, and excluded those without complete clinical data. They compared three prediction models: one based on proportional hazard regression analysis (COX), one using a back-propagation neural network (BPNN), and another employing a BPNN optimized with a genetic algorithm. The findings reveal that BPNN-based models significantly outperformed the COX model and are more effective for clinical risk assessments. However, further research is needed to refine sample size requirements and address limitations in genetic algorithm complexity.

Schäfer et al. [42] analyzed the risk factors for DFU and amputations using statistical models and ML techniques on a dataset of 246,705 diabetic patients from Danish national registries [43]. They employed COX proportional hazards (PHs) and Aalen Johansen models to estimate hazard rates and account for competing risks like mortality, identifying key risk factors. Researchers used ML models, including logistic regression (LR) and random forest (RF), to predict DFU and amputation occurrences at different timeframes. The RF model outperformed LR, demonstrating higher classification accuracy in risk prediction.

Reddy et al. [44] proposed the Extreme Learning Machine (ELM) and compared it with other machine learning models, including KNN, SVM with Gaussian kernel, and ANN, to predict foot ulcers. They used a dataset of 133 instances and 22 attributes sourced from the Figshare repository, focusing on the binary classification of DFU presence (positive or negative). After preprocessing the data for consistency, the ELM model, with a single hidden layer of 35 neurons and sigmoid activation, outperformed the other models across all evaluation metrics, outperforming KNN, SVM, and ANN.

Zhang et al. [19] developed an ANN model to predict the prognosis of DFUs using clinical and lower extremity computed tomography angiography (CTA) data. They utilized data from 203 patients collected from the WoundCareLog database [45] from 195 hospitals in China. Patients were categorized into two groups based on their Wagner scores, a grading system used to classify the severity of DFUs based on the depth of the ulcer and the presence of infection. The ANN model, implemented using the multilayer perceptron algorithm, consisted of three layers: an input layer with ten neurons corresponding to the predictive factors, a hidden layer with two neurons for non-linear data interactions, and an output layer that classified patients. The ANN achieved superior performance compared to LR.

Mousa et al. [18] evaluated two artificial intelligence models, an ANN and a DT, for predicting DFUs using medical data and foot images. A sample of 200 patients was included, 82 of whom had DFUs and 118 who did not, sourced from the National Institute of Diabetes and Endocrine Glands at Cairo University Hospital. The ANN model was designed with multiple layers: an input layer with nineteen nodes, two hidden layers with seven nodes each, and an output layer for binary classification: foot ulcer or non-foot ulcer. Images were preprocessed by resizing them to a consistent format, followed by feature extraction from the spatial and frequency domains using Fourier transform. Similarly, the DT model was constructed to classify patients based on medical data and image features. The results demonstrate that the ANN achieved superior performance compared to the DT.

Popa et al. [46] presented an ML methodology aimed at predicting mortality in patients afflicted with DFUs utilizing a dataset comprising 635 individuals who were admitted to the Diabetes, Nutrition, and Metabolic Diseases Clinic at Sf. Spiridon Emergency Clinical Hospital in Iasi, Romania. They developed two multilayer perceptron (MLP) models intended for predicting mortality at intervals of 5 years and 10 years after hospitalization. These models were architected with three hidden layers to enhance predictive performance. The design of the 5-year model included four neurons in the initial layer, a single neuron in the subsequent layer, and five neurons in the third layer. The 10-year model was slightly different, comprising five neurons in the first and second layers while incorporating four neurons in the third layer. Both computational models utilized ReLU activation functions and were subjected to training through a 10-fold cross-validation methodology to mitigate the risk of overfitting. The results indicate close levels of accuracy.

Kaushal et al. [47] examined the prediction of DF infections using deep learning techniques, employing a Kaggle dataset [36]. Critical features such as neuropathy, circulation issues, and skin thickness were extracted using MATLAB to enhance the prediction of diabetic foot ulcers. Participants were classified with and without foot ulcers, providing the basis for evaluating various classification algorithms. They compared neural networks, DT, regression model, and RF. Feature selection was a key component, with attributes extracted from foot images and medical data optimized to improve classifier performance. Models were assessed using metrics such as mean absolute error (MAE), root mean square error (RMSE), and relative absolute error (RAE). DT outperformed other methods, demonstrating the highest accuracy, making them the most effective model tested.

Liu et al. [16] introduced an ML model to predict amputation in patients with DFU. The study utilized a retrospective dataset of 150 patients from Beijing Shijitan Hospital. Researchers ensured that all included patients had DFU diagnoses above Wagner grade 1 and had undergone relevant diagnostic tests. The study employed the SVM algorithm to construct the model, chosen for its ability to handle complexity. The model development process followed multiple steps: (1) data preprocessing and variable selection using Lasso regression to identify critical predictors; (2) splitting the dataset into training and testing subsets using a five-fold cross-validation approach; (3) iterative model training and optimization to identify the best performing configuration; and (4) performance evaluation using metrics like the AUC. Researchers confirmed the model’s calibration using Hosmer–Lemeshow testing, demonstrating its reliability.

Tian et al. [48] presented an AI-based DF prediction model integrating Traditional Chinese Medicine (TCM) tongue diagnosis and Western clinical data. They used ResNet-50 to extract deep features from tongue images and a fully connected layer (FCL) to collect numerical data such as BMI. The study utilized tongue images, plantar hardness, clinical features, and laboratory data from 391 patients collected at the Second Affiliated Hospital of Tianjin University of Traditional Chinese Medicine. The model achieved high accuracy, outperforming the model without tongue images.

Hon et al. [17] explored ML techniques to predict the recurrence risk of DFUs in elderly diabetic patients, aiming to enhance prevention strategies. Using a dataset of 138 patients, the authors analyzed several risk factors, such as age and wound size. The study tested various ML models, including SVM, XGBoost, KNN, RF, and DT; among those models, the SVM achieved the highest accuracy. Data preprocessing involved outlier screening, feature integration, and correlation analysis to improve model robustness. Despite the model’s high performance, challenges such as potential biases in training data, limited interpretability, and the need for external validation were acknowledged.

Table 2 summarizes recent studies, highlighting AI models, data types, and performance metrics used for DFU prediction.

### 3.3. Segmentation

Marrero et al. [49] evaluated segmentation methods for DF monitoring using multimodal imaging such as infrared and depth for 37 participants. The researchers analyzed three segmentation models: UNet with Depth (UPD), Skin with Depth (SPD), and SegNet. UPD employs a CNN based on UNet architecture, which uses an encoder derived from VGG11 and an expanding decoder for pixel segmentation. The depth channel enhances accuracy by applying Random Sample Consensus to remove noise at boundaries. SPD is an unsupervised method that identifies skin pixels using thresholds across RGB, HSV, and YCbCr color spaces, optimized with depth data to remove outliers. SegNet uses a deep encoder–decoder architecture. The results show that UPD had reliable performance across varied conditions, making it the preferred model for practical use.

Bouallala et al. [21] introduced Double Encoder-ResUNet (DE-ResUNet), a deep learning model designed to segment diabetic foot thermal images by combining RGB and thermal data to improve segmentation accuracy. They utilized a dataset of 398 paired RGB and thermal images from the National Hospital Dos de Mayo in Peru. The researchers applied augmentation techniques like horizontal flipping, rotations, and contrast changes to address the limited sample size. The DE-ResUNet model combines the strengths of residual network and UNet architecture, adopting an encoder–decoder structure. Unlike a traditional UNet, DE-ResUNet uses two parallel encoders to process thermal and RGB images separately. DE-ResUNet outperformed all competitors, such as UNet and SegNet.

Huang et al. [50] proposed a system that integrates transfer learning and Fast R-CNN for object detection to enhance the diagnosis of diabetic foot wounds (DFWs). They employed transfer learning to harness pre-trained models, such as ResNet101 and Inception V2, for robust feature extraction and effective classification of wound types. Three Fast R-CNN modules, iNaturalist Species-trained (ResNet101), Kitti-trained (ResNet101), and Inception V2-coco, were used to evaluate performance. Among these, the Kitti-trained ResNet101 module demonstrated superior accuracy and performance. After detecting the wounds, they employed classical image processing methods like the GrabCut algorithm and SURF technique for segmentation. The dataset, provided by Taichung Veterans General Hospital, includes 727 images categorized into ulcers, sutures, and blood vessel blockages. To address data limitations, augmentation techniques such as flipping, rotation, and distortion were applied, expanding the dataset to 900 images per category for a total of 3600 images. The system also features a web-based interface, allowing medical professionals to upload and analyze images and view results.

Mahbod et al. [20] presented an approach for the automated segmentation of foot ulcers, addressing the extraction of morphological features from foot wounds. The authors proposed an ensemble of two encoder–decoder CNN architectures, UNet and LinkNet, integrated with pre-trained EfficientNet backbones, EfficientNetB1 for LinkNet and EfficientNetB2 for UNet, to enhance segmentation accuracy. To address limited data availability, the models were pre-trained on the Medetec dataset [51]. To optimize segmentation, the authors implemented five-fold cross-validation for generalizability, test time augmentation for prediction robustness, and result fusion through averaging predictions. The researchers tested the method on the Chronic Wound Dataset [52] and the extended FUSeg dataset [53]. This approach was ranked first in the FUSeg challenge.

Lan et al. [54] introduced FusionSegNet, a deep learning framework designed to improve the diagnosis of DFUs by integrating global foot features, e.g., skin wrinkles and abnormalities, with local wound features, e.g., wound depth, area, and location. The model operates in two stages: first, it utilizes a segmentation module to isolate wound areas using UNet pre-trained on the MICCAI FUSC2021 dataset [55]. In the second stage, a classification network based on ResNet-34 is employed to extract global and local features. The method is evaluated on the dataset collected by Shanghai Municipal Eighth People’s Hospital. Compared to existing methods, such as Inception-ResNet-v2, ResNet without CBAM, and DFUNet, FusionSegNet demonstrated the ability to distinguish DFUs from other chronic wounds.

Dhar et al. [56] introduced FUSegNet, an advanced deep learning model specifically developed to segment DFUs. FUSegNet is built on an encoder–decoder architecture. The encoder leverages EfficientNet-b7 to feature extraction. The decoder incorporates a parallel spatial and channel Squeeze-and-Excitation module, which combines additive and max-out operations. A modified version, x-FUSegNet, utilizes five-fold cross-validation to improve segmentation in complex backgrounds. Two datasets were used for evaluation: the Chronic Wound Dataset [52] and the FUSeg Challenge 2021 Dataset [53]. The x-FUSegNet achieved acceptable results in the FUSeg Challenge 2021, placing it at the top of the leaderboard.

Hresko et al. [57] introduced a method for diabetic foot ulcer segmentation by combining self-training with mixup augmentation. Researchers trained the neural network to generate weak labels using the self-training process. The model design utilizes an Attention UNet architecture, trained with dice and cross-entropy loss functions, and employs five-fold cross-validation for robustness. Researchers evaluated the method on three datasets: DFUC2022 [58], FUSeg [53], and RMIT [59], and demonstrated improvement in dice scores.

Table 3 summarizes recent studies, highlighting AI models, data types, and performance metrics used for DFU segmentation.

### 3.4. Detection

Khandakar et al. [60] presented an ML-based framework for detecting DFUs using thermogram images. They utilized the Plantar Thermogram Database, which Hernandez-Contreras et al. [61] originally published, which contains 167 foot-pair thermograms. They compared the traditional ML classifier, AdaBoost, with deep learning models, including MobileNetV2, DenseNet201, ResNet50, and InceptionV3. These deep learning models leverage transfer learning with ImageNet weights to enhance performance. The study’s key findings indicate that the AdaBoost classifier achieved the highest F1 score using optimized feature selection, while MobileNetV2 demonstrated strong performance when analyzing dual-foot thermograms. They suggested that AdaBoost is a viable option for deployment on mobile applications due to its low computational cost.

Yogapriya et al. [62] introduced DFINET, a CNN designed to enhance the automated detection of infections in DFU images. The dataset utilized is a DFU-Part (B) dataset [31]. The DFINET architecture features ten convolutional layers, five max-pooling layers, and five batch normalization layers, with ReLU and two fully connected layers, using parallel convolution filters to extract diverse features. The model uses the adaptive moment estimation (Adam) optimizer for weight updates and the SoftMax layer for binary classification. DFINET has promising results in infection recognition compared with other models such as GoogLeNet, VGG16, and AlexNet.

Thotad et al. [22] presented a method for detecting DFUs using the EfficientNet deep learning model. They utilized a dataset from the Kaggle repository [36]. The researchers applied data augmentation techniques such as rotation, flipping, and scaling to improve model training. EfficientNet is a CNN architecture designed to optimize feature extraction by balancing three key parameters: network width, depth, and image resolution. Unlike traditional CNNs, which typically scale these dimensions independently, EfficientNet employs a compound scaling method that adjusts all three dimensions simultaneously to achieve higher efficiency and performance. The comparative analysis demonstrated that EfficientNet significantly outperformed AlexNet, VGG16, DFUNet, and GoogleNet, which focus on individual dimensions like depth or width without comprehensive optimization.

Sarmun et al. [63] presented a deep learning system designed to enhance the detection and localization of DFUs. The system leverages two object detection models, YOLOv8m and Faster R-CNN ResNet101, combined using weighted bounding box fusion (WBF) for improved accuracy. YOLOv8m features a C2f backbone for efficient feature extraction. Faster R-CNN ResNet101 employs a two-stage detection framework, using a region proposal network for generating candidate bounding boxes and a classifier for refined predictions. The researchers evaluated this system using the DFUC2020 dataset [32] and validated it externally on the IEEE DataPort Diabetic Foot dataset [64]. Despite challenges such as distracting background objects in the validation set, the system achieved good results.

Biswas et al. [65] introduced FusionNet, a deep learning framework designed for DFU detection using multi-scale feature fusion and explainable artificial intelligence (XAI). The researchers preprocessed the DFU dataset from Kaggle [36] by applying Gaussian and median filtering, noise removal, and motion blur correction. Data augmentation techniques, such as rotation, flipping, zooming, and shearing, were applied to the training set to enhance diversity. FusionNet’s architecture combines three pre-trained CNN models, which are DenseNet201, VGG19, and NASNetMobile. DenseNet201 captures high-dimensional features, VGG19 focuses on fine-grained details, and NASNetMobile offers scalability and efficiency. Transparency is enhanced through integrated XAI algorithms, such as SHapely Adaptive Explanations (SHAP), Gradient-Weighted Class Activation Mapping (Grad-CAM), and Local Interpretable Model-Agnostic Explanations (LIME), which provide interpretable visualizations of predictions. The model achieved exceptional results, outperforming single-model approaches such as VGG19, DenseNet201, and NASNetMobile.

Giridhar et al. [23] introduced a deep learning approach for detecting DFUs using CNNs. The model utilizes the DFUC2021 dataset [34] and employs preprocessing techniques such as scaling, noise removal, and normalization to improve data quality and enhance model generalization. The architecture leverages DenseNet121, which incorporates dense blocks connected by transition layers that down-sample while preserving crucial information. The ReLU activation function adds non-linearity, while global average pooling minimizes spatial dimensions to reduce overfitting. Fully connected layers aggregate the extracted features for final classification into ischemia, infection, and none. The researchers pre-trained the model on the ImageNet dataset, allowing it to use transfer learning and demonstrate higher speed and precision compared to alternatives like EfficientNet-B3.

Table 4 summarizes recent studies, highlighting AI models, data types, and performance metrics used for DFU detection.

### 3.5. Generative AI in DFU

Hyun et al. [66] introduced a synthetic data generation system to address the scarcity of medical datasets for AI-based DF diagnosis. The framework consists of four modular stages. First, the Seed Data Generator creates transcutaneous oxygen pressure (TcPO₂) and foot temperature data, categorized into severity levels, e.g., critical ischemia or healthy, while glucose data are sourced from the UCI Diabetes Dataset [67]. Second, the Preprocessor applies two distinct methods: (i) statistical processing for seed data, ensuring it follows realistic distributions, and (ii) filtering of glucose data to retain only pre- and post-meal measurements for consistency. Third, the Augmentor introduces jittering, a time series data augmentation technique, to simulate realistic noise from wearable sensors, enhancing dataset variability. Finally, the Data Generator leverages NeuralProphet, a neural network-enhanced time-series model, to synthesize realistic trends and patterns in the data. The experimental results show that the system successfully generates diverse datasets that align with real-world patterns. However, a key limitation is its inability to synthesize multivariate data simultaneously, which may restrict its ability to model complex interdependencies.

Foomani et al. [68] tackled the challenge of limited access to electronic medical records (EMRs) for developing predictive models in chronic wound healing. The study introduced the EMR-Time-series Conditional Wasserstein Generative Adversarial Network (EMR-TCWGAN), a deep learning framework that synthesizes realistic EMR data while capturing temporal patterns from weekly patient follow-ups. The study employed RF classifiers to identify the most critical wound prognosis factors, which it then used in the GAN model. The researchers implemented conditional training strategies to enhance the generation of labeled data (healed vs. non-healed wounds). The model was evaluated using TSTR (test on synthetic, train on real), discriminative accuracy, and visualization techniques, demonstrating its potential to enhance wound healing predictions. This study derived its dataset from venous leg ulcer cases at AZH Wound and Vascular Centers, Milwaukee, WI, USA.

Basiri et al. [25] used diffusion models to generate synthetic images of DFU. Diffusion models introduce and iteratively remove noise from the data to recover the original data. The model applied Gaussian noise to the original image in the forward pass of diffusion. For the denoising step, a UNet architecture with attention layers was utilized to predict the noise distribution and project it back to the original DFU images. The model was trained on DFU-colored images from Goyal et al. [69]. The authors found that 70% of synthetic images were marked as real by clinicians. The researchers applied evaluation metrics such as Fréchet Inception Distance (FID) and Kernel Inception Distance (KID), but these metrics showed poor alignment with clinical assessments, suggesting the need for alternative approaches.

Jishnu et al. [24] developed Automatic Foot Ulcer Segmentation using Conditional GAN (AFSegGAN), a conditional GAN-based model for the segmentation of foot ulcers. The objectives included segmenting wound images and estimating morphological parameters, e.g., the wound area and perimeter. AFSegGAN incorporates a UNet generator for image synthesis and a CNN discriminator for segmentation. The model is integrated into a comprehensive wound management system comprising a patient portal and a physician portal connected via a cloud-based infrastructure. The patient portal enables users to upload wound images, which AFSegGAN processes to generate segmented wound masks and calculate metrics. Physicians can access detailed records and segmented images through the physician portal for analysis. The model utilizes images from the MICCAI 2021 Foot Ulcer Segmentation dataset [55] and applies augmentation techniques for robust performance. The findings demonstrate superior segmentation results, outperforming state-of-the-art models such as UNet-EffB2 [20] and DeepLabV3+SE [70].

El-Kady et al. [71] addressed the challenge of accurately diagnosing DFUs by integrating deep learning models. They compared the performance of a ResNet50 model and a hybrid ResNet50-GAN model for medical image analysis. The proposed framework involves six steps: data preprocessing, augmentation, feature extraction using ResNet50, synthetic data generation via GAN, hybrid model training, and evaluation. Techniques like GAN-generated image augmentation address the challenge of limited data, and ResNet50’s deep architecture extracts complex features, enhancing classification accuracy. The researchers obtained the dataset from the National Institute of Diabetes and Endocrinology in Egypt [72]. The study demonstrated the efficacy of integrating GANs to improve diagnostic precision and address data scarcity in medical imaging.

Table 5 summarizes recent studies, highlighting AI models, data types, and performance metrics used for generative AI in DFU.

### 3.6. Smartphone Applications for DFU

Kim et al. [6] applied ML models to predict the healing outcomes of DFUs by integrating clinical data from electronic health records (EHRs) with imaging features extracted from smartphone photographs. Researchers collected the dataset from 2291 clinical visits involving 381 DFUs from 155 patients treated at the Michigan Medicine Podiatry and Wound Clinic. They utilized handcrafted color and texture features and deep learning-based features from ResNet50 to analyze wound images. Podiatry staff manually segmented handcrafted imaging features, such as mean and standard deviation of color intensity, from wound photographs and processed them using MATLAB. In addition, deep learning features were extracted using ResNet-50; however, handcrafted features outperformed others, achieving an AUC of up to 0.794. The researchers trained ML models using RF and SVM algorithms to predict whether ulcers were healed or not healed. Despite some limitations, such as limited sample size and data imputation challenges, this work demonstrates the promise of combining ML with smartphone devices to improve DFU prediction.

Chan et al. [73] evaluated the reliability of the mobile application CARES4WOUNDS (C4W), Tetsuyu, Singapore, which is an AI-enabled tool for wound imaging and measurement. The study involved 28 patients with DFUs, and the main wound parameters were length and width. Using 547 wound images, the researchers measured parameters such as wound length, width, and area. The results demonstrated excellent intra-rater reliability (0.933–0.994) across three different devices and good inter-rater reliability when compared to manual wound measurements. Despite some limitations in wound depth detection, the study demonstrated that the C4W system is an effective tool for consistent wound monitoring.

Anisuzzaman et al. [74] presented an automated wound localization system using the YOLOv3 model, integrated into an iOS mobile application. The model is trained on the AZH Wound Database [75]. The system isolates wounds and their surrounding tissues in 2D images, removing irrelevant regions to enhance subsequent tasks like segmentation and classification. The YOLOv3 model was compared with the Single shot multibox detector (SSD) [76]; the performance evaluation showed that YOLOv3 achieved a mean Average Precision (mAP) of 93.9% on the AZH Wound Database, outperforming the SSD’s mAP of 86.4%. Retraining on the larger Wound Database further improved the mAP to 97.3%. The system’s robustness was validated on the Medetec Wound Database [77], demonstrating high generalizability. A lighter version of the model Tiny-YOLOv3 enables wound detection on mobile devices, ensuring ease of access for remote healthcare.

Cassidy et al. [77] conducted a proof-of-concept clinical evaluation of an AI-based system for automated DFU detection using smartphones. The study involved 81 diabetic patients recruited from two UK hospitals, who provided a total of 203 foot photographs via an Android mobile application with varying resolutions. The system utilizes a deep learning model based on Faster R-CNN for object detection and Inception-ResNetV2 for feature extraction, with transfer learning from the MS COCO dataset. Researchers trained the model on 1775 annotated DFU images and deployed it on a cloud platform for real-time analysis. During the clinical evaluation, clinicians captured images using the app, and the cloud-based AI system returned predictions with an average response time of 5.9 s per case. The model demonstrated high sensitivity and specificity. Clinical validation revealed excellent inter-rater and intra-rater reliability (Krippendorff’s α > 0.8), indicating strong agreement among clinicians.

Sendilraj et al. [78] developed DFUCare, a deep learning-based platform for the automated detection, classification, and monitoring of DFUs using smartphone images. The system employs YOLOv5s for wound localization and InceptionResNetV2 for infection and ischemia classification, integrating CIELAB and YCbCr color space segmentation for enhanced wound analysis. Researchers trained and validated the system using two benchmark datasets: DFUC2020 [32] and DFUC2021 [34]. In testing, DFUCare achieved an F1 score of 0.80 and a mAP of 0.861 for wound localization. A pilot study at the Postgraduate Institute of Medical Education and Research, India, demonstrated strong agreement with physician assessments, supporting the system’s clinical utility for remote wound monitoring.

Table 6 summarizes recent studies, highlighting AI models, data types, and performance metrics used for AI-based smartphone applications for DFU.

### 3.7. Evaluation Metrics and Datasets

The evaluation of AI models for DFU management metrics assesses various aspects of model performance. Accuracy is the most critical metric for all models, offering a clear measure of the model’s reliability by identifying the proportion of correct predictions among all predictions. Following this, the F1 score is important for its ability to balance precision and recall, making it particularly important in handling imbalanced datasets. For segmentation tasks, the dice score and IoU are crucial. The dice score evaluates the overlap between predicted and ground truth regions, while the IoU quantifies the precision of wound area identification by measuring the overlap between predicted and actual segmentations. For prediction tasks, researchers use the MAE and RMSE to assess the magnitude of errors in continuous predictions. While the MAE provides the average magnitude of errors, the RMSE gives greater weight to larger errors, making it useful when larger deviations are critical. Additionally, the RAE normalizes the MAE by comparing it against how a baseline model performs. The AUC is critical for classification models as it measures how well a model distinguishes between classes across different thresholds. These metrics provide a multidimensional framework for evaluating the effectiveness of AI models in improving DFU management. Table 7 presents all the equations for the metrics.

Researchers benefit from the presentation of available datasets as it supports the development of AI-based solutions for DFUs and ensures study reproducibility. Various datasets advance machine learning and deep learning in DFU management. The DFU-Part (B) dataset [31] provides infection and ischemia foot images, featuring 9870 ischemia patches and 5892 bacterial infection patches. The DFUC2020 dataset [32] offers 4000 diverse images for training and testing, while the DFUC2021 dataset [34] focuses on infection and ischemia detection with 15,683 image patches, which researchers classify into four classes: control (healthy skin), infection, ischemia, and a combination of both conditions (infection and ischemia). The DFUC2022 dataset [58] includes 2000 fully annotated images intended to support segmentation tasks. The Medetec dataset [51] offers 152 wound images with segmentation masks for DFU detection. The Kaggle repository [36] consists of four folders: Original Images, Patches, TestSet, and TransferLearning Image. The Original Images folder includes 493 images, while the Patches folder contains 1055 images. The TestSet folder includes 167 images, while the TransferLearning Image folder comprises 959 images. The Chronic Wound Dataset [52] contains 1109 images from 889 patients. Lastly, the FUSeg dataset [53], an extended version of the Chronic Wound Dataset, features 1210 foot ulcer images that researchers gathered over two years for segmentation research. These datasets provide essential data that enhance AI model development.

The performances of the reviewed models are summarized in Table 1, Table 2, Table 3, Table 4, Table 5 and Table 6. However, since the models are trained and tested on different datasets and for various tasks, providing a standardized performance comparison is challenging. For instance, the FUSeg dataset is specifically designed for segmentation tasks, while other datasets focus on classification or prediction. The aim of this section is to highlight the most commonly used metrics for evaluating the performance of AI-based solutions for DFUs.

## 4. Discussion

AI has transformed the management of DFUs by delivering significant advancements in classification, prediction, segmentation, and detection. Traditional approaches, such as visual assessments and manual measurements of wound dimensions, vary depending on the clinician’s experience. On the other hand, AI-driven tools enable highly accurate early detection of ulcers through advanced image processing techniques and machine learning models that extract features, such as color intensity and texture metrics.

Generative AI has played a critical role in overcoming one of the most critical challenges in medical AI, which is data scarcity. The limited availability of diverse datasets has hindered the development and generalizability of robust AI models, particularly in DFU management, where privacy concerns often restrict the collection of high-quality medical data. By creating realistic synthetic datasets through advanced techniques such as GANs and diffusion models, generative AI provides a solution to this problem. These synthetic datasets allow researchers and developers to validate models while reducing dependence on large, costly real-world data collections.

Integrating smartphone applications into DFU management helps solve key challenges in traditional healthcare delivery, such as frequent hospital visits and limited specialist access. These applications enable monitoring, measuring wound dimensions, and tracking healing progress. Clinicians can remotely review patient data and provide timely recommendations, making these tools particularly valuable for the early detection of DFUs.

### 4.1. Roles of AI in DFU Management

AI significantly contributes to managing DFUs through various tasks, including classification, prediction, segmentation, detection, generative AI, and smartphone applications. Classification models, such as CNNs, are highly effective in categorizing DFUs into conditions like infection or ischemia, providing essential diagnostic support to clinicians. Similarly, prediction models, using algorithms such as ANNs, forecast key outcomes like the likelihood of healing or the risk of amputation, equipping healthcare providers with actionable insights for early interventions.

Segmentation, another significant task, focuses on delineating wound boundaries using models like UNet, enabling the precise measurement of wound size, depth, and progression. Wound measurements are essential for tracking healing and guiding treatment strategies. Detection involves identifying DFUs within complex image backgrounds and serves as an essential first step before classification or segmentation. Models like EfficientNet excel in this task, making them particularly valuable in remote monitoring scenarios.

Despite its potential, researchers have explored generative AI less frequently in DFU research. However, it addresses a data scarcity challenge. Generative AI leverages models like GANs and diffusion models to synthesize realistic datasets, enhancing the performance, reliability, and generalizability of classification and prediction models. Finally, smartphone applications provide an efficient platform for integrating AI into healthcare management. These applications help perform real-time wound detection, classify wound types, and measure wound dimensions, reducing the need for frequent clinical visits.

### 4.2. Challenge

AI offers significant potential to improve DFU management, but several practical challenges persist in reality. First, collecting and labeling DFU image data are time-consuming and require considerable clinical expertise. Second, inconsistencies in how DFUs are classified lead to variations between categories. Third, DFU datasets lack standardization due to differences in factors such as camera distance, image orientation, and lighting conditions. Lastly, variations in patient characteristics, including ethnicity, age, sex, and foot size, create diverse and uneven datasets [79].

AI-based smartphone applications hold great promise but encounter challenges that impede their broader adoption. One major issue is the lack of interpretability and trust in AI systems. Many models, especially those utilizing deep learning, appear as ‘black-box’ systems, leading to skepticism among healthcare providers and patients due to their lack of transparency. Another challenge is the generalization of AI models across diverse populations [80]. These models perform poorly on datasets that differ from their training data [81]. Ethical concerns, including biases in predictions, further complicate the integration of AI into healthcare. Overcoming these challenges will require more transparent, inclusive, and ethically governed AI systems to enable broader acceptance of smartphone-based healthcare applications [80].

### 4.3. Future Direction

There are several areas where the application of AI and generative AI can be prioritized to advance DFU management. Although various AI-based solutions for DFU have been proposed in the literature, researchers must conduct more studies to clinically validate them in real-world applications. Longitudinal studies that track patient outcomes over time can assess the efficacy and safety of these AI-based solutions. Another important aspect is the acceptability and usability of such solutions. Researchers should conduct studies to assess their usability for both patients and clinicians, as well as their impact on the patient experience. One of the most critical aspects is gaining the confidence of healthcare professionals, which hinges on improving the interpretability of AI models. To address this, developing XAI frameworks in the context of DFU is essential. Researchers could apply existing XAI methods, such as SHAP, Grad-CAM, and LIME, to enhance model transparency. Although these methods have been utilized in some DFU models, such as DFU_XAI [82] and FusionNet [65], researchers still need to conduct a quantitative assessment of their efficiency in DFU. By making AI-driven decisions more interpretable, these frameworks allow clinicians to validate and trust AI-generated recommendations. Another critical focus is the need to increase dataset diversity, a key factor in overcoming biases and improving the generalizability of AI models. Addressing this challenge requires collaboration among researchers, healthcare institutions, and AI developers to create more inclusive datasets. Additionally, synthetic data generation using generative AI techniques, such as GANs and diffusion models, can play a pivotal role in augmenting real-world data. Addressing these priorities will not only accelerate the adoption of AI in healthcare but also ensure effective solutions for managing DFUs.

## 5. Conclusions

In conclusion, this study emphasizes the progress achieved through the integration of AI into DFU management. The review explored developments in AI-driven classification, prediction, segmentation, and detection in several areas, such as enabling early intervention and supporting personalized patient care. The study also highlighted the role of generative AI in addressing the issue of data scarcity and how smartphone applications contribute to enabling remote monitoring.

This study identified several challenges that researchers must overcome to advance the clinical adoption of AI in DFU management. Researchers must enhance interpretability, expand access to diverse datasets, and streamline the time-intensive process of collecting and labeling DFU image data. Additionally, inconsistencies in DFU classification and a lack of standardization in datasets further complicate model development.

Future research should focus on overcoming these limitations by advancing explainable AI models. Furthermore, the use of generative AI techniques represents a promising solution for generating synthetic datasets to enhance AI model training and evaluation. By addressing these challenges, the integration of AI in DFU management can be optimized to establish effective solutions for global healthcare systems.

## Figures and Tables

**Table 1:** Summary of AI-based classification models for DFUs.

Ref.	Year	Study Aim	Data Type	Model Used	Code Availability	Hyperparameter	Training Protocols	Evaluation Metrics
[13]	2020	Classify DFUs and healthy skin	Image	DFU_QUTNet + SVM	Not provided	Batch size: 32, learning rate: 0.5, dropout rate: 0.5, number of layers: 58	Training: 80%, testing: 20%	F1 score: 1. DFU_QUTNet: 94.5% 2. AlexNet: 89.1% 3. VGG16: 90.9% 4. GoogleNet: 92.9% 5. DFUNet: 93.1%
[30]	2020	Classification and localization of ischemia and infection in DFUs	Image	CNN, YOLOv2	Not provided	Learning rate: 0.001, epochs: 40	Training: 50%, testing: 50%	Accuracy: Ischemia: 97% Infection: 99%
[15]	2023	Classify ischemia and infection in DFUs	Image	ResNet50	Not provided	Batch size: 32, learning rate: 0.001, momentum: 0.8, epochs: 30	Training: 80%, validation: 10%, testing: 10%	Accuracy: 1. ResNet50: Ischemia: 99.49% Infection: 84.76% 2. AlexNet: Ischemia: 83.56% Infection: 83.22% 3. VGG16: Ischemia: 98.58% Infection: 79.32% 4. GoogleNet: Ischemia: 99.65% Infection: 79.66% 5. DenseNet: Ischemia: 99.30% Infection: 83.20% AUC: ResNet50: Ischemia: 99.96% Infection: 94.16%
[33]	2023	Classify none, infection, ischemia, or both in DFUs	Image	DFU-SIAM + KNN	Not provided	Batch size: 8, learning rate: 10 × 10^−6^, epochs: 40	K-fold validation (K = 5)	Accuracy: 95%
[14]	2023	Classify ischemia and infection in DFUs	Image	EfficientNet	Not provided	Batch size, learning rate	Training: 70%, validation: 15%, testing: 15%, 5-fold cross-validation, transfer learning	Accuracy: 1. EfficientNet: Infection: 97% Ischemia: 99% 2. DenseNet121: Infection: 94% Ischemia: 96% 3. ResNet50: Infection: 87% Ischemia: 94% 4. Inception V3: Infection: 86% Ischemia: 98% 5. VGG16: Infection: 85% Ischemia: 95%
[35]	2023	Classify abrasion foot sores and ischemic diabetic foot sores	Image	Vgg-19 with UNet++	Not provided	Batch size: 32, learning rate: 0.05 (decayed by 0.1 every 10 epochs), momentum: 0.8, epochs: 150	Training: 70%, validation: 20%, testing: 10%, transfer learning	1. Vgg-19 with UNet++: Accuracy: 99.05% F1 score: 99.04% AUC: 0.996 2. Inception-v3: Accuracy: 95.52% F1 score: 95.53% AUC: 0.930 3. MobileNet: Accuracy: 96.73% F1 score: 96.94% AUC: 0.990
[37]	2023	Classify DFUs as normal or abnormal	Image	AWSg-CNN	Not provided	Batch size, learning rate	Training: 80%, testing: 20%	Accuracy, F1 score, AUC: 99%
[38]	2023	Classify foot ulcer images as normal or abnormal	Image	DRNN, PFCNN	Not provided	Batch size: 32, learning rate: 0.01 (decayed by 0.1 every 25 epochs), momentum: 0.9, epochs: 2000	Training: 50%, testing: 50%	Accuracy: 99.32%
[39]	2024	Real-time classification of DFUs	Image	DFU_FNet, DFU_TFNet	Not provided	Batch size: 32, learning rate: 0.001, epochs: 100	Transfer learning	1. AlexNet: Accuracy: 89.11% F1 score: 88.1% 2. VGG16: Accuracy: 90.37% F1 score: 90.9% 3. GoogleNet: Accuracy: 91.93% F1 score: 92.9%. 4. DFU_FNet + SVM: Accuracy: 94.71% F1 score: 94.5% 5. DFU_TFNet: Accuracy: 99.81% F1 score: 99.25%
[40]	2025	DFU classification	Image	RL, CPPN, SVM, ELM, ResNet50	Not provided	Learning rate: 0.1, discount factor = 0.9	Reinforcement learning	Classification accuracy: 93.75% Clustering Efficiency: Cluster 1: 71–88% Cluster 2: 85–97% Cluster 3: 90–98% Cluster 4: 93.5–98.2%

**Table 2:** Summary of AI-based prediction models for DFU.

Ref.	Year	Study Aim	Data Type	Model Used	Code Availability	Hyperparameter	Training Protocols	Evaluation Metrics
[41]	2020	Predict amputation and survival risks in patients with DF	Numerical	COX Regression-Based, BPNN, BPNN+GA	Not provided	Learning rate: 0.001, transfer function: trainlm and purelin	Training set: 160 patients; test set: 40 patients	AUC: 1. COX Regression: Amputation: 0.557 Death: 0.635 2. BPNN: Amputation: 0.924 Death: 0.712 3. BPNN+GA: Amputation: 0.891 Death: 0.712
[42]	2021	Predict risk of DFU and amputation	Numerical	LR, RF	Not provided	Not mentioned	Training: 75%, testing: 25%	Accuracy: LR: 95%
[44]	2021	Predict presence of DFUs in patients	Numerical	ELM, SVM, ANN	Not provided	Hidden neurons: 35 (ELM), 10 (ANN); K value: 9 (KNN); SVM kernel: Gaussian	Training: 80%, testing: 20%	Accuracy: 1. ELM: 96.15% 2. SVM: 92.31% 3. ANN: 84.62%
[19]	2022	Predict prognosis of DFUs	Numerical	ANN, LR	Not provided	ANN: Learning rate: 0.4, max training time: 15 min, hidden layer: hyperbolic tangent function, output layer: SoftMax function	Training: 60%, testing: 20%, holdout sample: 20%	1. ANN: Accuracy: 91.6% AUC: 0.955 2. LR: Accuracy: 82.2% AUC: 0.874
[18]	2023	Predict positive or negative DFUs	Image and Numerical	ANN and DT	Not provided	ANN: Input features: 19, Hidden layers: 2	Training: 75%, testing: 25%, k-fold cross-validation (k = 10)	Accuracy 1. ANN: 97% 2. DT: 93%
[46]	2023	Predict mortality in patients with DFU	Numerical	MLP	Not provided	5-year model: 3 hidden layers (4-1-5 neurons); 10-year model: 3 hidden layers (5-5-4 neurons)	Training: 80%, testing: 20%, 10-fold cross-validation	1. 5-Year Model: Training Accuracy: 77.2% Test Accuracy: 72.4% 2. 10-Year Model: Training Accuracy: 75.9% Test Accuracy: 70.9%
[47]	2024	Predict diabetic foot infections and ulcers	Numerical	Neural networks, DT, RF, regression model	Not provided	Not mentioned	Model comparison using multiple classifiers	1. RF: MAE: 0.0077 RMSE: 0.0233 RAE: 1.6878% 2. DT: MAE: 0.0005 RMSE: 0.0040 RAE: 0.0800% 3. Neural Networks: MAE: 0.0065 RMSE: 0.0310 RAE: 1.4285% 4. Regression Model: MAE: 0.0144 RMSE: 0.023 RAE: 3.1635%
[16]	2024	Predict amputation risk in patients with DFU	Numerical	SVM	Not provided	5-fold cross-validation	Data are divided into training sets and test sets by using 5-fold cross-validation	Accuracy: 82.4% AUC: 0.89
[48]	2024	Predict DF in patients with T2DM using Traditional Chinese Medicine and Western medicine	Numerical and image	ResNet-50	Not provided	Pretrained on ImageNet, last three convolution layers removed, FCL for feature extraction	311 images for training, 80 for testing, 5 random seed sets	Accuracy: With tongue image 0.95 Without tongue image: 0.92
[17]	2024	Predict recurrence risk of DFUs in elderly diabetic patients	Numerical	SVM	Not provided	C = 20, degree = 2, gamma: scale, kernel type: linear	Training: 70%, testing: 30%, 5-fold cross-validation	Accuracy: SVM: 93% XGBoost: 82% KNN: 82% RF: 82% DT: 79%

**Table 3:** Summary of AI-based segmentation models for DFUs.

Ref.	Year	Study Aim	Data Type	Model Used	Code Availability	Hyperparameter	Training Protocols	Evaluation Metrics
[49]	2021	Evaluate segmentation methods for DF monitoring	Image	UPD, SPD, and SegNet	Not provided	Learning rate: 0.1, momentum: 0.9, epochs: 150	Training: 50 images, testing: 24 images	1. UPD: DICE: 95.35 ± 0.40% IoU: 91.11 ± 0.72% 2. SPD: DICE: 95.24 ± 0.52% IoU: 90.93 ± 0.95% 3. SegNet: DICE: 93.30 ± 2.91% IoU: 87.57 ± 5.01%
[21]	2022	Segment diabetic foot images that fuse thermal and RGB data	Image	DE-ResUNet	Not provided	Learning rate: 0.01, momentum: 0.9, weight decay: 0.0005, ResNet-50 encoder	Training: 50%, validation: 25%, testing: 25%	IoU: 1. DE-ResUNet: 97% 2. SegNet: 95% 3. UNet: 95%
[50]	2022	Image segmentation to enhance diagnosis of DFWs	Image	Fast R-CNN	Not provided	Not mentioned	Training: 30%, testing: 10%, transfer learning, training iterations: 100,000	Accuracy: Wound image detection: 90% 1. Inception V2-coco: 87% 2. Kitti- ResNet101: 88% 3. Species-ResNet101: 89%
[20]	2022	Segmentation of foot ulcers	Image	UNet and LinkNet	https://github.com/masih4/Foot_Ulcer_Segmentation (accessed on 18 November 2024)	Learning rate: 0.001 (reduced by 90% every 25 epochs), batch size: 4, epochs: 80, loss function: dice and focal loss	5-fold cross-validation	Dice Score: UNet and LinkNet: 88.80% UNet with ASPP: 82.29%
[54]	2023	Diagnose DFUs by integrating segmentation of wound areas with classification	Image	FusionSegNet	Not provided	Learning rate: 1 × 10^−5^, batch size: 16, epochs: 100, loss function: binary cross-entropy	5-fold cross-validation	Accuracy: 1. FusionSegNet: 95.78% 2. Inception-ResNet-v2: 84.96% 3. DFUNet: 86.45%
[56]	2024	Segmentation of DFUs	Image	FUSegNet	https://github.com/mrinal054/FUSegNet (accessed on 25 November 2024)	Learning rate: 1 × 10^−4^, batch size: 2, loss function: dice and focal loss, epochs: 200	5-fold cross-validation	Dice Score: 1. x-FUSegNet: 89% 2. UNet with HarDNet68: 87% 3. Stacked UNets: 86%
[57]	2024	DFU segmentation using self-training and mixup augmentation	Image	Attention UNet	Not provided	Learning rate: 0.001, batch size: 8, epochs: 750, loss function: dice and cross-entropy	Training: 70%, validation: 10%, testing: 20%, 5-fold cross-validation	Dice Score: 1. DFUC 2022: 0.711 2. FUSeg: 0.859

**Table 4:** Summary of AI-based detection models for DFU.

Ref.	Year	Study Aim	Data Type	Model Used	Code Availability	Hyperparameter	Training Protocols	Evaluation Metrics
[60]	2021	Detection of DFUs	Image	AdaBoost, MobileNetV2, DenseNet201, ResNet50, InceptionV3	Not provided	Transfer learning	Training: 80%, testing: 20%, 5-fold cross-validation	F1 Score: 1. AdaBoost: 97.75% 2. MobileNetV2: 92.50% 3. DenseNet201: 94.01% 4. ResNet50: 93.41% 5. InceptionV3: 93.71%
[62]	2022	Detection of infection in DFU images	Image	DFINET	Not provided	Learning rate: 0.0001, batch size: 16, epochs: 30, loss function: binary cross-entropy	Training: 70%, validation: 20%, testing: 10%	F1 score: 1. DFINET: 92.12% 2. GoogLeNet: 76.39% 3. VGG16: 83.54% 4. AlexNet: 77.54%
[22]	2023	Detection of DFUs	Image	EfficientNet	Not provided	EfficientNet (depth, width, resolution)	Training: 60%, validation: 20%, testing: 20%	F1 score: 1. AlexNet: 89.1% 2. VGG16: 91.0% 3. DFUNet: 93.3% 4. GoogleNet: 93.0% 5. EfficientNet: 99.0%
[63]	2024	Detection and localization of DFUs	Image	YOLOv8m and Faster R-CNN ResNet101	Not provided	Learning rate: 0.001, epochs: 100–150	Training: 80%, validation: 10%, testing: 10%, transfer learning	F1 score: 0.780 mAP@0.5: 0.864
[65]	2024	Detection of DFUs using XAI	Image	FusionNet	Not provided	Learning rate: 0.0001, batch size: 32, epochs: 50, loss function: binary cross-entropy	Training: 70%, validation: 10%, eesting: 20%, transfer learning	1. VGG19: Accuracy: 86.7% F1 Score: 85.4% 2. DenseNet201: Accuracy: 97.6% F1 Score: 97.7% 3. NASNetMobile: Accuracy: 77.3% F1 Score: 72.1% 4. FusionNet: Accuracy: 99.05% F1 Score: 99.08%
[23]	2024	Detection and classification of DFUs	Image	DenseNet-201	Not provided	Learning rate: 0.01, batch size: 30, epochs: 7	Transfer learning	1. DenseNet-201: Accuracy: 98% F1 score: None: 98% Infection: 97% Ischemia: 98% 2. EfficientNet-B3: Accuracy: 98% F1 score: None: 100% Infection: 97% Ischemia: 97%

**Table 5:** Summary of generative AI in DFU models for DFU management.

Ref.	Year	Study Aim	Data Type	Model Used	Code Availability	Hyperparameter	Training Protocols	Evaluation Metrics
[66]	2021	Synthetic data generation system	Numerical	NeuralProphet	Not provided	Epochs: 100	Training: 60%, validation: 20%, testing: 20%	Accuracy of binary classification: 100%
[68]	2022	Address limited EMR access by generating synthetic data	Numerical	EMR-TCWGAN	Not provided	Batch normalization	4-fold cross-validation	AUC: 0.875 Accuracy: 77.98%
[25]	2023	Generate synthetic DFU images	Image	Diffusion model	Not provided	Learning rate: 1 × 10^−4^ (decaying), batch size: 32, epochs: 500	Gaussian noise	FID: 0.73 KID: 0.14
[24]	2023	Automatic foot ulcer segmentation	Image	AFSegGAN	Not provided	Learning rate: 0.002, batch size: 16	Training steps = epochs × batch per epoch	IoU: 1. AFSegGAN: 99.07% 2. UNet-EffB2: 85.01% 3. DeepLabV3+SE: 92.4%
[71]	2024	Enhance accuracy of DFU diagnosis	Image	ResNet50, ResNet50-GAN	Not provided	Learning rate: 0.001, batch size: 4 (ResNet50), 8 (ResNet50-GAN), epochs: 100	8-fold cross-validation	Accuracy: ResNet50: 76% ResNet50-GAN: 84% F1 score: ResNet50: 75% ResNet50-GAN: 84%

**Table 6:** Summary of AI-based smartphone applications for DFU.

Ref.	Year	Study Aim	Data Type	Model Used	Code Availability	Hyperparameter	Training Protocols	Evaluation Metrics
[6]	2020	Predict healing outcomes of DFUs	Numerical	RF, SVM	Not provided	RF: 2000 hyperparameter combinations (bootstrapping, tree criteria, depth, splits, PCA components); SVM: 2028 combinations (C, gamma, PCA components)	Training: 75%, testing: 25%, 3-fold cross-validation	Handcrafted image features: 1. RF: Accuracy: 0.811 F1 Score: 0.760 2. SVM: Accuracy: 0.784 F1 Score: 0.794
[73]	2021	Validate reliability of CARES4WOUNDS for wound measurement DFUs	Image and Numerical	C4W app	Not provided	Not mentioned	manual vs. AI-based wound measurement	Intra-rater reliability > 0.9
[74]	2022	Wound localization system in DFUs	Image	YOLOv3, tiny-YOLOv3	https://github.com/uwm-bigdata/wound_localization (accessed on 19 December 2024)	Learning rate: 0.001, batch size: 8, epochs: 273	Training: 4050 images	F1 Score: 0.949
[77]	2023	Automated detection of DFUs	Image	Faster R-CNN with Inception-ResNetV2	Not provided	Transfer learning	Training: 1775 images	F1 Score: 94%
[78]	2024	Detection, classification, and monitoring of DFUs	Image	YOLOv5s (localization), InceptionResNetV2 (classification)	Not provided	YOLOv5s: learning rate: 0.01, epochs: 30	Training: 1800 images, testing: 200 images, 10-fold cross-validation	Classification accuracy: Infection: 79.76% Ischemic: 94.81%

**Table 7:** The equations of performance evaluation metrics for AI models in DFU management.

Metric	Equation	Parameters
Accuracy	Accuracy = $\frac{TP+TN}{TP+TN+FP+FN}$	TP: True Positive, TN: True Negative, FP: False Positive, FN: False Negative
F1 Score	F1 Score = 2 × $\frac{Precision\times Recall}{Precision+Recall}$	$Precision=\frac{TP}{TP+FP}$, $Recall=\frac{TP}{TP+FN}$
Dice	Dice _image_ = $\frac{1}{N}\sum_{i=1}^{N}\frac{2TPi}{2TPi+FPi+FNi}$	TP: True Positive, FP: False Positive, FN: False Negative
IoU	IoU _image_ = $\frac{1}{N}\sum_{i=1}^{N}\frac{TPi}{TPi+FPi+FNi}$	TP: True Positive, FP: False Positive, FN: False Negative
AUC	AUC = $\frac{1}{2}\left(\frac{TP}{TP+FN}\right)+\left(\frac{TN}{TN+FP}\right)$	TP: True Positive, TN: True Negative, FP: False Positive, FN: False Negative
MAE	MAE = $\frac{1}{n}$ $\sum_{i=1}^{n}\left|yi-\hat{yi}\;\right|$	yi: actual value, $\hat{yi}$: predicted value, n: total number of data points
RMSE	RMSE = $\sqrt{\frac{1}{n}}\sum_{i=1}^{n}\left(yi-\hat{yi}\right)$^2^	yi: actual value, $\hat{yi}$: predicted value, n: total number of data points
RAE	RAE = $\frac{\sum_{i=1}^{n}\left|yi-\hat{yi}\;\right|}{\sum_{i=1}^{n}\left|yi-\bar{y}\;\right|}$, $\bar{y}=\frac{1}{n}\sum_{i=1}^{n}yi$	yi: actual value, $\hat{yi}$: predicted value, n: total number of data points, $\bar{y}$: mean of all actual values

## Data Availability

Not applicable.

## Abbreviations

The following abbreviations are used in this manuscript:

AI	artificial intelligence
AFS	abrasion foot sores
AUC	area under the receiver operating characteristic curve
ANN	artificial neural network
BPNN	backpropagation neural network
BIM	body mass index
CNN	convolutional neural network
DM	diabetes mellitus
DF	diabetic foot
DFU	diabetic foot ulcer
DFS	diabetic foot sores
DFW	diabetic foot wounds
DT	decision tree
DICE	dice similarity coefficient
DFUC	diabetic foot ulcer challenge
EHR	electronic health record
EMR	electronic medical record
ELM	extreme learning machine
FCL	fully connected layer
FID	Fréchet inception distance
GA	genetic algorithm
GAN	generative adversarial network
Grad-CAM	gradient-weighted class activation mapping
HbA1C	hemoglobin 1C
IoU	intersection over union
KNN	k-nearest neighbor
KID	kernel inception distance
LR	logistic regression
LIME	local interpretable model-agnostic explanations
ML	machine learning
mAP	mean average precision
MAE	mean absolute error
MLP	multilayer perceptron
NB	naive bayes
PVD	peripheral vascular disease
PAD	peripheral artery disease
RF	random forest
RL	reinforcement learning
ROC	receiver operating characteristic
ReLU	rectified linear unit
RAE	relative absolute error
RMSE	root mean squared error
SNN	Siamese neural network
SVM	support vector machine
SHAP	shapley additive explanations
T2DM	type 2 diabetes mellitus
TcPO₂	transcutaneous oxygen pressure
XAI	explainable artificial intelligence
